# Rapid redistribution of agricultural land alters avian richness, abundance, and functional diversity

**DOI:** 10.1002/ece3.5713

**Published:** 2019-10-06

**Authors:** Stephen Pringle, Ngoni Chiweshe, Peter R. Steward, Peter J. Mundy, Martin Dallimer

**Affiliations:** ^1^ Durrell Institute of Conservation and Ecology University of Kent Canterbury UK; ^2^ Forest Resources and Wildlife Management National University of Science and Technology Bulawayo Zimbabwe; ^3^ Sustainability Research Institute School of Earth and Environment University of Leeds Leeds UK

**Keywords:** biodiversity conservation, land sharing, land sparing, land‐use change, smallholder farming, Zimbabwe

## Abstract

The conversion of natural, or seminatural, habitats to agricultural land and changes in agricultural land use are significant drivers of biodiversity loss. Within the context of land‐sharing versus land‐sparing debates, large‐scale commercial agriculture is known to be detrimental to biodiversity, but the effects of small‐scale subsistence farming on biodiversity are disputed. This poses a problem for sustainable land‐use management in the Global South, where approximately 30% of farmland is small‐scale. Following a rapid land redistribution program in Zimbabwe, we evaluated changes in avian biodiversity by examining richness, abundance, and functional diversity.

Rapid land redistribution has, in the near term, resulted in increased avian abundance in newly farmed areas containing miombo woodland and open habitat. Conversion of seminatural ranched land to small‐scale farms had a negative impact on larger‐bodied birds, but species richness increased, and birds in some feeding guilds maintained or increased abundance. We found evidence that land‐use change caused a shift in the functional traits of the communities present. However, functional analyses may not have adequately reflected the trait filtering effect of land redistribution on large species.

Whether newly farmed landscapes in Zimbabwe can deliver multiple benefits in terms of food production and habitat for biodiversity in the longer term is an open question. When managing agricultural land transitions, relying on taxonomic measures of diversity, or abundance‐weighted measures of function diversity, may obscure important information. If the value of smallholder‐farmed land for birds is to be maintained or improved, it will be essential to ensure that a wide array of habitat types is retained alongside efforts to reduce hunting and persecution of large bird species.

## Graphical abstract

Land‐use change following rapid land redistribution has varied impacts on avian diversity.

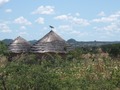

## 1 INTRODUCTION

A fundamental driver of global biodiversity loss is the conversion of natural habitats to agriculture (Hooper et al., 2012; Vié et al., 2009). It is estimated that the current rate of global human population growth will lead to a 59%–98% rise in food demand between 2005 and 2050 (Valin et al., 2014). Demand for food increases land scarcity, which is thought to drive the conversion of natural vegetation to agriculture. This theory has led to much debate, often based on concepts of land sharing and land sparing, to examine the trade‐offs between food production and biodiversity conservation (Phalan, Onial, Balmford, & Green, 2011; Tscharntke et al., 2012). For instance, evidence from Ghana, India, Uganda, and Borneo suggests that “land‐sparing” landscapes, with segregated areas of agriculture and nonfarmed habitat, are more likely to meet both goals as compared to “land‐sharing” landscapes, where agriculture and natural habitats are interspersed (Edwards et al., 2010; Hulme et al., 2013; Phalan et al., 2011). However, the environmental costs of agriculture are often overlooked and the impacts on functional biodiversity across farmed landscapes are often poorly understood (Tscharntke et al., 2012). Furthermore, the theoretical benefits of land sparing have rarely been demonstrated in the field (Fischer et al., 2014). Partial trade‐off analyses that provide support for land sparing ignore real‐world complexity in terms of the scale or type of farming undertaken (for instance, smallholder‐farmed landscapes form the backbone of global food security (Samberg, Gerber, Ramankutty, Herrero, & West, 2016)). These analyses also fail to account for regional variations in how agriculture expands and the associated implications for persistence of biodiversity (Fischer et al., 2014; Tscharntke et al., 2012). Even if land sparing can reduce habitat loss within a system, retention of biodiversity may be less than expected if mechanisms to prevent anthropogenic disturbance are lacking (Barlow et al., 2016; Fischer et al., 2014).

Land‐use change is generally driven by increasing demand for agricultural commodities and land scarcity. However, it may also be driven by policies designed to address major societal issues, such as poverty and fair access to land, through socioeconomic change (Chappell et al., 2013). Major socioeconomic changes are generally accompanied by rapid land‐use change in rural areas. In sub‐Saharan Africa, historical patterns of inequitable land distribution are key factors linking impoverished rural livelihoods, food security, and food sovereignty (Clover & Eriksen, 2009). Several countries in eastern, central, and southern Africa have implemented land tenure reform policies (Clover & Eriksen, 2009). In Namibia, semiarid savanna redistributed to land reform beneficiaries is often farmed in small units by settlers with limited farming experience (Lohmann, Falk, Geissler, Blaum, & Jeltsch, 2014). Assessment of the ecological implications of Namibian land resettlement by small‐scale farmers suggests it is sustainable in the short term, with no savanna degradation due to bush encroachment (Lohmann et al., 2014). However, the effects of land tenure changes over longer timescales may be less sustainable (Dougill et al., 2016). In Zimbabwe, the Fast‐Track Land Reform Programme (FTLRP) led to the resettlement of eight million hectares between 2000 and 2007 (Moyo & Matondi, 2008). Over the same period, total Zimbabwean agricultural output decreased by 44%, largely due to lower large‐scale commercial production (Clover & Eriksen, 2009). In many areas of Zimbabwe, newly resettled rural communities now engage in subsistence agriculture on marginal lands, creating new social, economic, and ecological challenges, such as habitat degradation and loss (Fakarayi, Mashapa, Gandiwa, & Kativu, 2015). In parallel with changes in land tenure and agricultural practice, by 2007, it was estimated that Zimbabwean wildlife had declined by 60% in national parks, and 50%–80% in conservancies and game farms (Degeorges & Reilly, 2007). Zimbabwe may illustrate an example of national policies resulting in a shift from land sparing to land sharing, where the displacement of large‐scale commercial production by more mixed smallholder agriculture has resulted in declines in biodiversity. However, more information is needed to draw robust conclusions. Currently, there is limited information on taxonomic or functional biodiversity responses to conversion of natural habitats to small‐scale farming in Africa. This restricts our ability to determine trade‐offs in the relationship between food production and biodiversity conservation (Tscharntke et al., 2012).

Global avian abundance has declined by around a quarter since agriculture became widespread (Gaston, Blackburn, & Goldewijk, 2003). This decline is strongest in intensively farmed areas (Newton, 2004). Where forest is converted to agriculture, diverse or small‐scale agricultural landscapes may help to mitigate declines in taxonomic measures of bird biodiversity, such as species richness or diversity (Frishkoff et al., 2014; Plexida & Sfougaris, 2015), but this is inconsistently observed (Sinclair, Mduma, & Arcese, 2002; Sinclair, Nkwabi, Mduma, & Magige, 2014; Tscharntke et al., 2008). Even in diverse agricultural landscapes, land‐use change differentially affects bird species according to their traits. For example, human activity in tropical and subtropical forests reduces the abundance and occurrence of long‐lived, large, nonmigratory, primarily frugivorous or insectivorous forest species (Newbold et al., 2013). It has also been shown to reduce avian functional diversity (Edwards, Edwards, Hamer, & Davies, 2013) and avian phylogenetic diversity (Frishkoff et al., 2014). As the impacts of land‐use change on biodiversity can be context‐specific, it is critical to consider more than just taxonomic measures of biodiversity. To provide insights into trade‐offs between sub‐Saharan African farming systems and biodiversity, we assess the effects of land‐use change on both taxonomic diversity and functional diversity of avian communities in a Zimbabwean context. The study system is a land redistribution area where commercial livestock farming with large seminatural areas (land sparing) has been redistributed and replaced by small‐scale farming where agricultural production and unfarmed habitats are intermixed (land sharing). Specifically, we test the hypotheses that: (a) the redistribution of land results in decreased avian species richness and diversity; (b) the redistribution of land results in a decline in large‐bodied species; and (c) changes in (a) and (b) will be reflected in a shift in the functional traits of communities present.

## 2 METHODS

### 2.1 Study system and survey method

**Figure 1:**
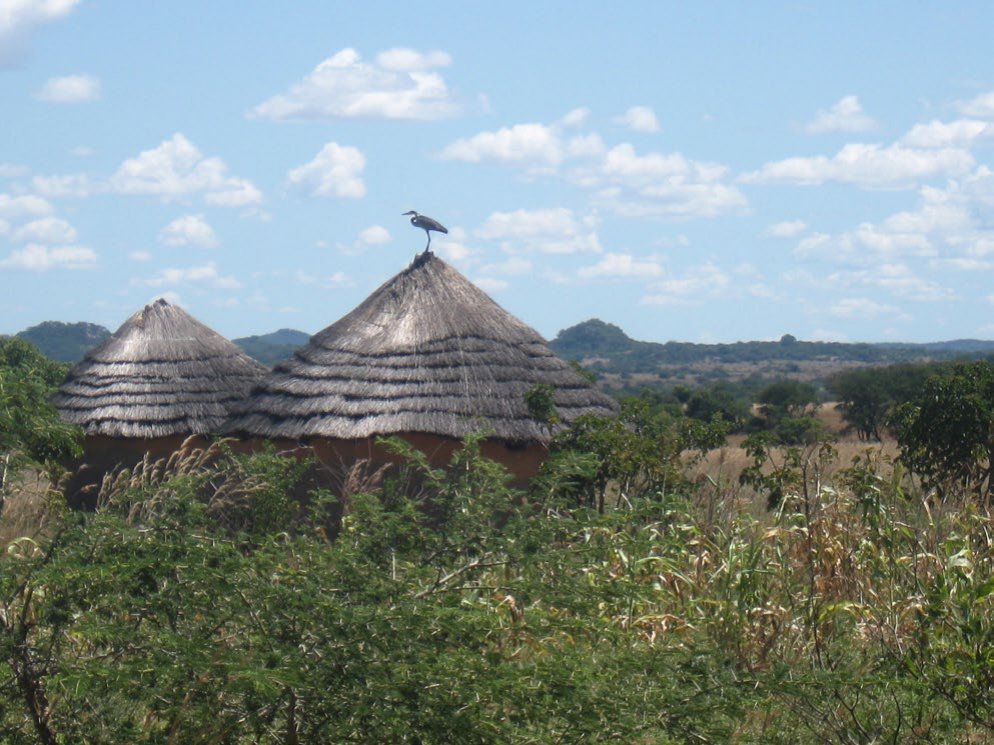
Homesteads in the resettled lands of the study area in central southern Zimbabwe. Photo: Ngoni Chiweshe

**Figure 2:**
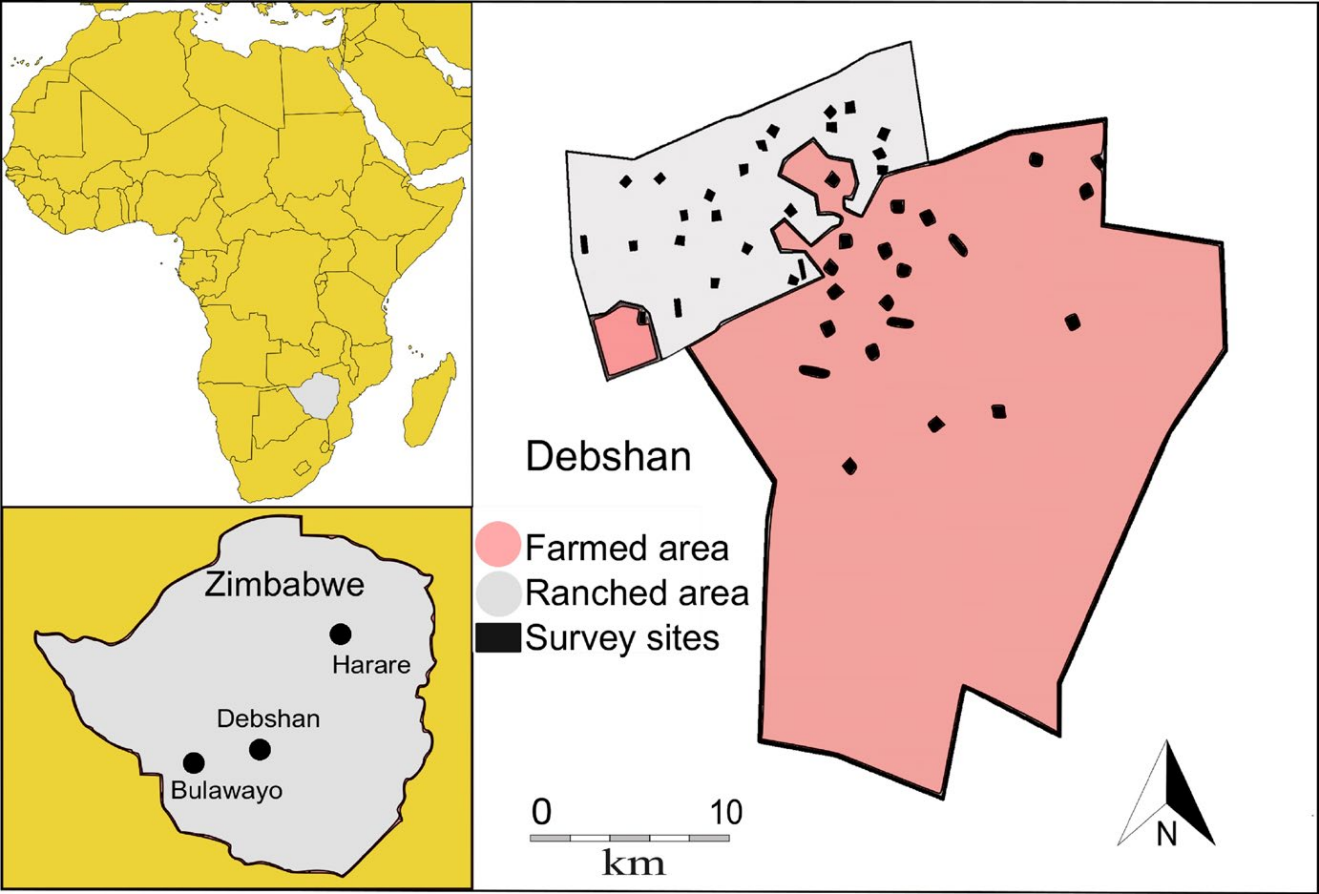
The study area (Debshan) in Africa and location within Zimbabwe showing the location of the survey transect sites

**Figure 3:**
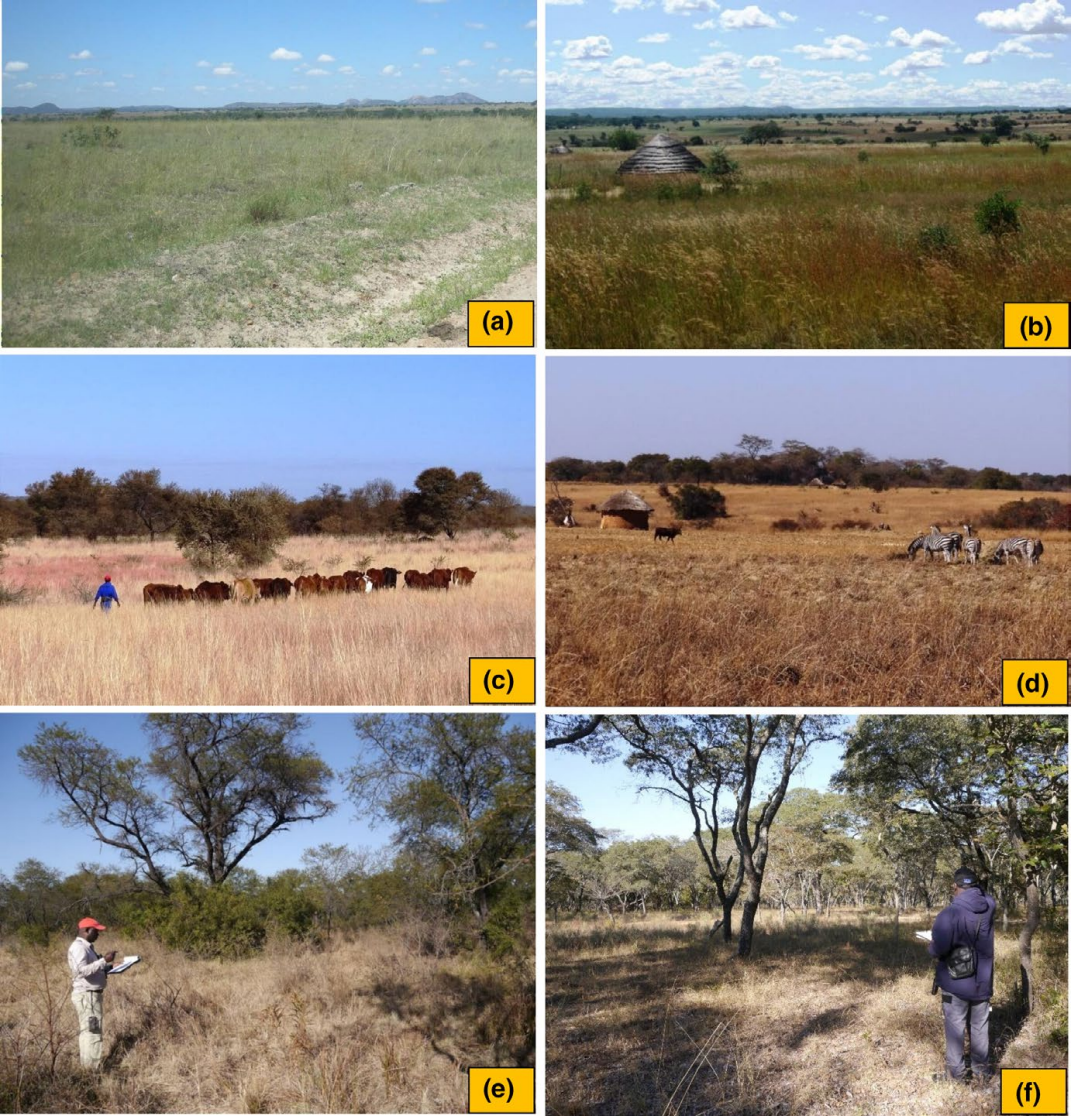
Typical transect sites in mid‐summer (a‐b) and mid‐winter (c‐f). In both ranched (a, c, f) and farmed areas (b, d, e), livestock and game animals graze freely throughout open habitats (a‐d), acacia woodlands (e), and miombo woodlands (f). In summer, small arable fields adjacent to homesteads in the farmed open habitats (b) are cropped at a subsistence level to provide the annual food supplies for resettled families. Photos: Ngoni Chiweshe (a‐b); Martin Dallimer (c–f)

The study area is located on a 91,000‐ha area of central southern Zimbabwe (29°34′E, 20°04′S) that was formerly in private ownership, and originally used for cattle and game ranching (Figures 1, 2, 3). When in private ownership, the entire ranch was characterized by areas of open habitat, miombo (dominated by *Brachystegia* spp.) and acacia woodland (dominated by *Acacia* spp.). During the FTLRP, the Zimbabwean government acquired around 72% of the ranched area for redistribution, leaving the remaining land to the original owners. In a rapid resettlement program during 2001–2002, around 3,000 families were moved into allocated 5‐ to 6‐ha plots spread throughout the redistributed area. Retention of land by original owners and the continuation of land management practices on that land were unusual during the FTLRP. The study area therefore offers a unique insight into how the avian community has responded to shifts in land tenure and land use. Land that did not change ownership is still utilized entirely for cattle and game ranching (hereafter referred to as “ranched areas”). The redistributed land is now used for smallholder mixed livestock and arable subsistence farming (farmed areas). At the time of this study, lands in both ownership categories continued to contain extensive areas of open habitat, miombo and acacia woodland (Figure 3). The open habitat within the farmed area contained both small cropped fields close to homesteads, and grassland; in the ranched area, there was only grassland. In both farmed and ranched areas, livestock are grazed through all habitat types, but feed mainly in the open habitat grasslands and fallow fields.

Bird surveys were carried out along linear transects, which were selected with random start points and orientations from Google Earth images. Transects were chosen to be as homogenous as possible between sites, but with no other selection criteria. The relative abundance of different habitat types in ranched and farmed areas was taken into account in selecting sites. Thus, a greater number of open sites were surveyed, as this was the main habitat type. Wooded sites were categorized as acacia (*Acacia* spp. dominant) or miombo woodlands (*Brachystegia* spp. dominant). In total, 45 sites were surveyed: 23 ranched (acacia *n* = 5, miombo *n* = 7, open *n* = 11) and 22 farmed (acacia *n* = 5, miombo *n* = 6, open *n* = 11). The distances (mean; *SD*; closest) between ranched and farmed sites of the same habitat type were as follows: acacia, (16.1; 3.2; 3.5) km; miombo, (13.3; 1.8; 3.4) km; and open, (11.2; 1.1; 3.6) km.

Bird surveys were carried out shortly after sunrise, or before sunset, in fine weather with good visibility. When two sites were counted on the same day, they were selected from different habitat types, and situated as far apart as possible. Each site was surveyed twice, once in winter (June 2012), when resident birds dominate and Palaearctic migrants are absent, and once in summer (January 2013). Two 600‐m parallel transects, spaced 300 m apart, were walked at constant slow speed; mean transect duration was 118 min (*SD* 16.7). In total, 108 km of transects was surveyed. Data recorded for each bird species observation were as follows: the number of individuals, the angle of deviation from the transect direction, and the distance from the observer (measured using a Leica LRF1200 rangefinder). Care was taken to avoid double‐counting birds and to exclude those overflying. Only observations within 100 m of transects were considered to represent birds using the survey site. While carrying out transects, the number of people, homesteads, dogs, cut trees, crops, and cattle were also recorded as indicators of the level of human activities.

### 2.2 Data analyses: richness, density, and biomass

A database of traits was compiled using data from publications listing the relevant Zimbabwean subspecies (Brown, Urban, & Newman, 1982; Fry, Keith, & Urban, 1988, 2000; Fry & Keith, 2004; Keith, Urban, & Fry, 1992; Urban, Fry, & Keith, 1986, 1997). Identification at subspecies level is of importance in selecting the correct mensural trait data for our subsequent analyses. The database comprised seven traits for each species: five measurements of morphology (average adult body mass, and lengths of wing, tail, bill, and tarsus), feeding guild (frugivore, granivore, insectivore, nectarivore, or predator), and migratory behavior (resident, intra‐African migrant, or Palaearctic migrant). Species were also classified into primary feeding guilds (De Graaf, Tilghman, & Anderson, 1985), which added an omnivore category to the above five guilds (Table S1).

Species richness (SR) was estimated using EstimateS 9.1.0 software (Colwell, 2013) to analyze individual‐based count data by land use (ranched or farmed), season (winter or summer), and habitat type (open habitat, acacia woodland, or miombo woodland). In estimating SR, no corrections were made to take account of species‐ or habitat‐dependent variations in detection probabilities. Species accumulation, interpolation (rarefaction), and extrapolation curves were used to evaluate sampling adequacy and to calculate Chao1 estimators of species richness. The ecological significance of differences in species richness between land uses was assessed by comparing 95% confidence intervals (CIs) and effect sizes.

Bird population densities, corrected for detection probabilities, were estimated using Distance 7.1 software (Thomas et al., 2010). Conventional Distance Sampling mode was used, with two modeling options: half normal functions with Cosine series expansion and uniform functions with simple polynomial series expansion (Buckland et al., 2001). The most parsimonious model solution was chosen using Akaike's information criterion (Buckland et al., 2001). Density estimates, stratified by land use, season, and habitat, were calculated from the counts for: (a) all species grouped by migratory behavior; (b) all species grouped by primary feeding guild; (c) all species grouped into adult body mass ranges (as proxies for bird sizes); (d) common species (i.e., those with >60 detections); and (e) rarer species (<60 detections) grouped by prominence (defined in Table S2). Biomass estimates for every species were calculated by multiplying species‐level population density estimates by the average adult body mass of each species. These species‐level biomass estimates were subsequently grouped into biomasses of each feeding guild.

The similarity of avian assemblages according to land use, stratified by habitat, was visualized by nonmetric ordination plots using PAST 3.10 software (Hammer et al., 2001). All species' count data were Log_10_ (*x* + 1)‐transformed before running Bray–Curtis distance analyses adjusted for missing data. Bray–Curtis residual distances and projection geometry were used to generate ordination plots onto the first two axes of three‐dimensional fits. For each feeding guild, a correlation coefficient (represented as a vector from the origin) was calculated between the guild and the ordination score. One‐way analyses of similarity (ANOSIM) tests using the Bray–Curtis index were run with 9,999 permutations.

### 2.3 Functional traits' analyses

The impact of land‐use change on functional traits' diversity was analyzed using the population density estimates, stratified by land use and habitat (with summer and winter counts combined), and the database of bird traits (Table S1). Traits were selected to reflect key aspects of resource usage that drive ecosystem functions (Şekercioğlu, 2006). Body metrics were used to represent rate of resource consumption (mass), foraging mode and behavior (bill and tarsus), and flight range for resource access and dispersal (wing and tail). Prior to analyses, values for each morphometric trait were standardized to a mean value of zero and unit standard deviation, thus weighting these traits equally. Morphological metrics were defined using continuous scales of millimeters or grams. Feeding guilds are relevant in terms of ecosystem services (e.g., seed dispersal, pollination), population control and resource removal (e.g., insectivore and predator–prey capture), and nutrient recycling (e.g., fecal deposition). Binary feeding guilds were each assigned a 1/5 weighting; this ensured that these traits were not unduly weighted in those species assigned to more than one guild (Laliberté et al., 2014). Abundance‐weighted functional trait indices for the avian communities for each land use and habitat combination were calculated in R software (R Development Core Team, 2016) using the “fundiv” package. Trait–species distance calculations were performed using Gower's similarity function to allow incorporation of trait types of mixed scales (Podani & Schmera, 2006).

The functional diversity (FD) index is closely related to species richness (Petchey & Gaston, 2006) and does not fully represent the multifaceted aspects of community functional diversity (Villéger, Mason, & Mouillot, 2008). To alleviate these limitations, three independent functional indices are often used to quantify the relationships between species' abundances, their traits, and trait variability (Villéger et al., 2008). For each community, we calculated functional diversity (FD), functional evenness (Feve), and functional divergence (Fdiv). These are, respectively: the dispersion of an assemblage of species in trait space; the regularity of distribution in trait space of species weighted by their abundances; and the proportion of total abundance in the assemblage formed by those species with the most extreme trait values (Mouillot, Graham, Villéger, Mason, & Bellwood, 2013; Petchey & Gaston, 2006; Villéger et al., 2008). The indices are constrained to the range 0–1. Values for a fourth index, functional richness, were not calculated, as species richness was below the exponential of the number of traits included in the analyses (Villéger et al., 2008).

## 3 RESULTS

While walking transects, we observed that farmed sites (*n* = 22) showed substantially higher impacts from human use than those that were ranched (*n* = 23). The following indications of impact are for all habitats combined: higher numbers of people (totals of 193 vs. 10 on all farmed sites and all ranched sites, respectively); homesteads (100 vs. 4); and dogs (64 vs. 1). In addition, cut trees were more common (evidence seen on 21 farmed sites vs. 1 ranched site). All ranched sites were uncultivated, whereas diverse crops including maize, sugar beans, groundnuts, roundnuts, finger millet, and gourds were present in 20 farmed sites. Similar total numbers of cattle were observed in each land‐use type (509 vs. 495).

**Figure 4:**
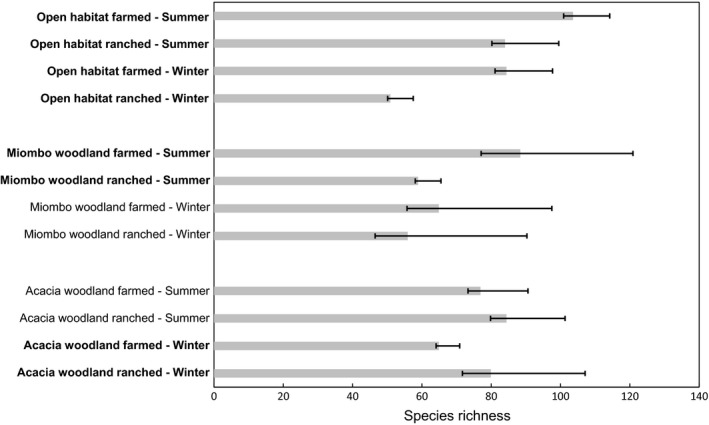
Avian species richness (SR) by habitats in farmed and ranched sites by season. SR values are individual‐based rarefaction estimates calculated from count data using EstimateS V9.1.0 software. Error bars represent 95% confidence intervals. Legends in bold are those where the difference in SR between farmed and ranched sites in the same season and habitat type shows an effect size > 1

In ranched sites, a total of 3,066 individuals of 136 species were counted, compared with 3,702 individuals of 155 species in farmed sites. Rarefaction curves of birds recorded in each season, habitat, and land‐use type (Figure S1) indicated that adequate sampling was achieved. The potential ecological significance of changes in SR in relation to land use was assessed in terms of 95% CIs and where effect sizes > 1 (Figure 4). Species richness estimates indicated that, in austral winter, open habitats in the farmed land held significantly more species than ranched open habitats, while the reverse was true for acacia woodlands. During austral summer, species richness in open habitats and miombo woodlands in farmed areas was higher than in these habitats on ranched land.

Densities of resident and migrant birds varied according to habitat and land use (Table 1). With one exception (miombo woodlands, in summer), farmed habitats of all types held significantly higher densities of resident birds in both winter and summer than were present during these seasons in ranched habitats. In contrast, intra‐African migrants showed a significant preference for ranched, compared with farmed, open habitat in summer. Palaearctic migrants were present only in summer; these birds showed no land‐use affinities.

**Table 1:** Bird densities by season, habitat, and land use for resident and migrant species, based on counts corrected for detection probabilities

Season	Migratory behavior	Acacia woodland				Miombo woodland				Open habitat			
		Ranched (120 ha)		Farmed (120 ha)		Ranched (168 ha)		Farmed (144 ha)		Ranched (264 ha)		Farmed (264 ha)	
		Birds/ha	95% CI	Birds/ha	95% CI	Birds/ha	95% CI	Birds/ha	95% CI	Birds/ha	95% CI	Birds/ha	95% CI
Summer	Resident	6.32	5.29–7.55	7.59	6.27–9.18	3.70	2.74–5.00	3.75	2.96–4.74	3.84	3.23–4.57	4.97	4.23–5.85
	Effect size	1.75				0.08				2.74			
	Intra‐African migrant	0.22	0.11–0.45	0.37	0.13–1.08	0.28	0.09–0.92	0.13	0.07–0.26	0.33	0.17–0.64	0.08	0.04–0.17
	Effect size	0.56				0.61				2.15			
	Palaearctic migrant	0.83	0.49–1.38	0.84	0.53–1.31	0.51	0.27–0.97	0.48	0.22–1.04	1.21	0.88–1.65	1.02	0.72–1.45
	Effect size	0.04				0.12				0.86			
Winter	Resident	5.38	4.37–6.61	8.66	6.85–10.95	1.87	1.37–2.57	4.77	3.83–5.93	1.83	1.47–2.28	4.92	3.88–6.25
	Effect size	3.50				6.06				6.10			
	Intra‐African migrant	0	0	0	0	0	0	0	0	0.07	0.02–0.29	0.81	0.17–3.90
	Effect size	na				na				0.66			
	Palaearctic migrant	0	0	0	0	0	0	0	0	0	0	0	0
	Effect size	na				na				na			

Note

In winter, intra‐African migrants were present only in the open habitat, and Palaearctic migrants were absent. Effect size (ES) values for each category of migratory behavior are calculated for bird densities in the same season and habitat type, but different land uses. Values that may indicate ecologically significant differences in densities are highlighted (blue, ES = 0.8–1.0; red, ES > 1.0).

In general, densities stratified by primary feeding guild (Table 2) were maintained or increased in farmed land that had previously been ranched. This was observed for granivores and insectivores in all habitat types; for frugivores, omnivores, and predators in open habitat; and for omnivores in miombo woodland. This trend was reversed for nectarivores in miombo woodland, and for omnivores and predators in ranched acacia woodland.

**Table 2:** Estimated bird densities for winter and summer combined, categorized by primary feeding guild, habitat and land use, based on counts corrected for detection probabilities

Feeding guild	Acacia woodland				Miombo woodland				Open habitat			
	Ranched (120 ha)		Farmed (120 ha)		Ranched (168 ha)		Farmed (144 ha)		Ranched (264 ha)		Farmed (264 ha)	
	Birds/ha	95% CI	Birds/ha	95% CI	Birds/ha	95% CI	Birds/ha	95% CI	Birds/ha	95% CI	Birds/ha	95% CI
Frugivore	1.02	0.75–1.40	1.10	0.80–1.53	0.39	0.25–0.62	0.46	0.30–0.71	0.35	0.24–0.52	0.46	0.33–0.63
Effect size	0.39				0.57				1.27			
Granivore	0.79	0.57–1.09	2.11	1.40–3.18	0.43	0.30–0.63	1.36	0.96–1.93	0.86	0.59–1.25	1.41	1.07–1.86
Effect size	3.29				4.43				2.56			
Insectivore	3.40	2.86–4.05	3.85	3.23–4.59	1.87	1.47–2.40	2.23	1.77–2.82	2.07	1.75–2.42	2.80	2.43–3.23
Effect size	1.27				1.26				3.65			
Nectarivore	0.16	0.09–0.27	0.15	0.07–0.30	0.32	0.17–0.61	0.14	0.07–0.26	0.09	0.04–0.18	0.06	0.04–0.11
Effect size	0.15				1.54				0.81			
Omnivore	1.37	1.03–1.81	1.05	0.69–1.60	0.35	0.25–0.49	0.58	0.41–0.82	0.38	0.28–0.53	0.84	0.60–1.16
Effect size	1.26				2.35				3.61			
Predator	0.05	0.03–0.11	0.01	0.00–0.04	0.05	0.02–0.14	0.16	0.05–0.49	0.04	0.02–0.08	0.08	0.05–0.13
Effect size	1.65				0.93				1.73			

Note

Effect size (ES) values for each feeding guild are calculated for bird densities in the same habitat type, but different land uses. Values that may indicate ecologically significant differences in densities are highlighted (blue, ES = 0.8–1.0; red, ES > 1.0).

Avian biomass stratified by habitat and land use showed numerous significant differences according to feeding guilds, with differences in population densities of larger birds being a major factor (Table 3). Despite being present in relatively low numbers (which precluded detailed analyses of their data at species level), density variation (or absence) of six large species of omnivores and predators dominated the total avian biomass in each habitat and land use (Table S3).

**Table 3:** Estimated avian biomass for winter and summer combined, categorized by primary feeding guild, habitat and land use, based on counts corrected for detection probabilities

	Acacia woodland		Miombo woodland		Open habitat		Major factors determining change in biomass
	Ranched	Farmed	Ranched	Farmed	Ranched	Farmed	
Frugivore (*n*)	163	153	70	81	145	206	
Frugivore biomass (g/ha)	94.8	118.1	45.4	41.1	37.0	48.0	
Frugivore biomass (95% CI)	81.7–110.1	103.1–135.3	33.1–62.9	30.6–56.8	30.2–46.2	40.4–57.8	
Effect size	2.81		0.50		2.27		
Granivore (*n*)	122	213	66	157	224	569	
Granivore biomass (g/ha)	40.4	73.4^#^	33.7	51.0^#^	43.1	60.2^#^	More waxbills, doves, bishops, widowbirds^#^
Granivore biomass (95% CI)	33.3–49.4	61.9–87.6	23.1–48.9	35.3–75.8	31.6–59.0	51.6–71.0	
Effect size	5.44		1.68		2.47		
Insectivore (*n*)	493	432	314	316	853	1,022	
Insectivore biomass (g/ha)	145.7	138.9	58.4	67.9	94.7	123.9**	Most small–medium size species increased**
Insectivore biomass (95% CI)	118.9–189.1	115.9–167.7	44.5–78.8	53.3–91.3	81.4–112.7	102.0–162.0	
Effect size	0.36		0.85		1.92		
Nectarivore (*n*)	24	19	31	17	20	26	
Nectarivore biomass (g/ha)	2.0	2.1	1.7	1.2	0.6	0.9	
Nectarivore biomass (95% CI)	1.8–2.3	1.9–2.4	1.5–2.0	1.0–1.4	0.5–0.7	0.8–1.0	
Effect size	0.65		3.78		5.88		
Omnivore (*n*)	241	119	81	105	176	233	Kori Bustard (*n* = 3) only in ranched area^†^
Omnivore biomass (g/ha)	721.5^†^	55.5^††^	47.5	44.3	108.5	45.0^††^	Far fewer francolins, spurfowl, guineafowl^††^
Omnivore biomass (95% CI)	592.1–895.7	48.3–63.7	33.9–67.1	32.8–61.4	103.8–115.0	39.2–53.7	
Effect size	10.59		0.34		16.21		
Predator (*n*)	10	0	16	8	17	26	Detection probability = 1 from Distance 7.1*
Predator biomass (g/ha)	85.5	0.0^‡^	221.8	41.6^‡^	28.7^‡‡^	69.1	No White‐backed Vulture, African Fish Eagle^‡^
Predator biomass (95% CI)	85.5–85.5*	0.0–0.0	221.8–221.8*	23.3–74.7	28.7–28.7	52.1–91.6	No Black‐headed Heron, secretarybird^‡‡^
Effect size	na		15.83		4.98		

Note

Effect size (ES) values for each feeding guild are calculated for avian biomasses in the same habitat type, but different land uses. Values that may indicate ecologically significant differences in densities are highlighted (blue, ES = 0.8–1.0; red, ES > 1.0).

**Figure 5:**
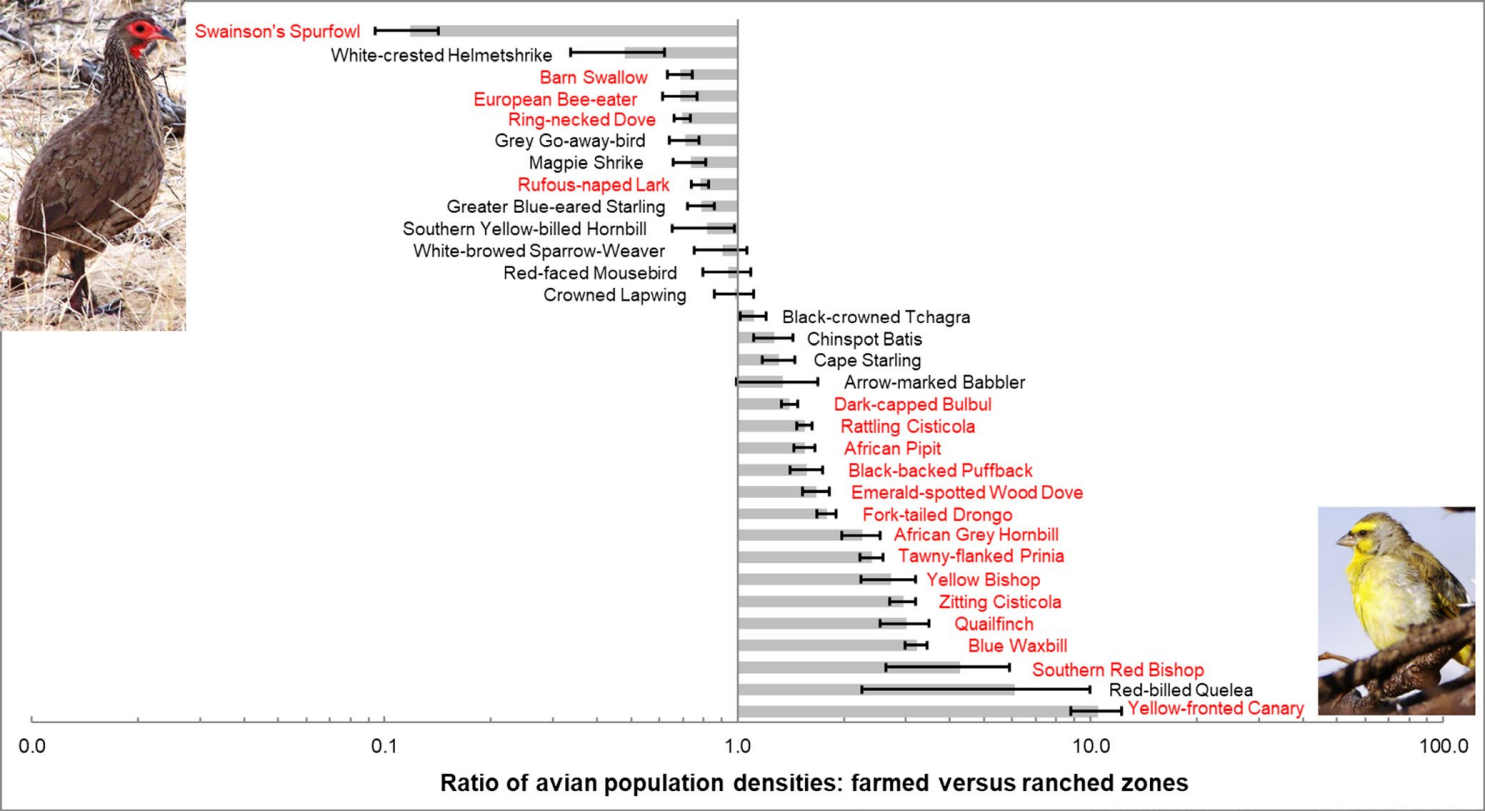
Ratios of densities, for all seasons and habitats combined, of common (*n* > 60 individuals) species recorded in farmed sites compared with ranched sites. Ratios > 1 indicate higher density in farmed sites. Error bars represent 95% confidence intervals around the mean ratio values. Species' labels in red are those where the density difference between farmed and ranched sites shows an effect size > 1. Photos: Stephen Pringle (left: Swainson's Spurfowl; right: Yellow‐fronted Canary)

**Figure 6:**
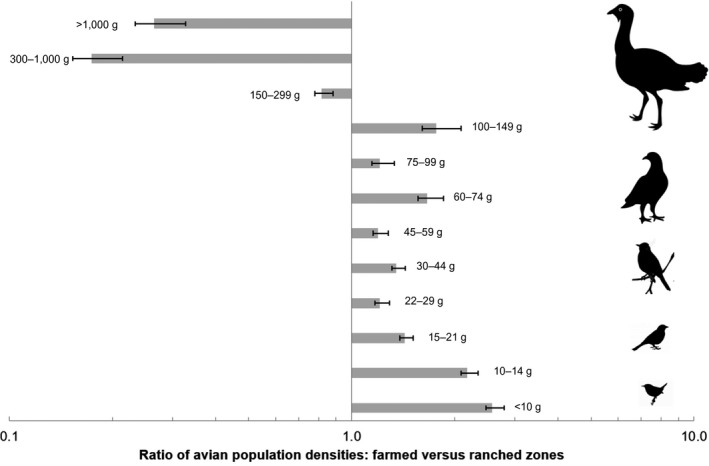
Ratios of densities, for all seasons and habitats combined, of species grouped by mean adult body mass, in farmed sites compared with ranched sites. Each horizontal bar represents the ratio of densities for all species within the body mass range shown in grams; each range contains approximately equal numbers of species. Ratios > 1 indicate higher density in farmed sites. Error bars represent 95% confidence intervals around the mean ratio values. For all mass ranges, the density difference between farmed and ranched sites shows an effect size > 1

No breeding colonies, or roosts, of any bird species were encountered in our transect counts. With the exception of Red‐billed Quelea *Quelea quelea*, no other species recorded in our counts occurred in large flocks (>30 individuals). Even in this species, only 164 individuals were counted across all transects in both seasons combined. Of the 179 bird species recorded, 32 were present in large enough numbers (>60 individuals) to permit analyses of population density differences by land‐use category at species level (Figure 5). These common birds represented 67.6% of the total number of individuals counted. Fourteen species occurred at higher densities (effect sizes > 1) in farmed sites, five species showed the opposite pattern (effect sizes > 1), and densities were unchanged (effect sizes < 1) in 13 species. In terms of body mass dependence (Figure 6), larger birds with mass >150 g were negatively affected by land‐use change, while smaller birds were present at higher densities (all effect sizes > 1).

**Figure 7:**
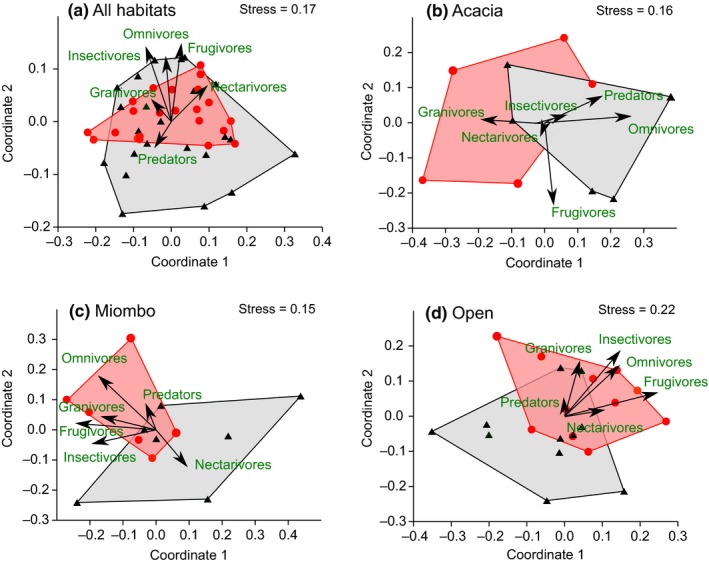
Nonmetric ordination plots of the avian communities for all seasons combined, showing (a) all habitats combined, (b) acacia woodland, (c) miombo woodland, and (d) open habitat. Each symbol represents a transect site in the farmed (filled circles) and ranched (filled triangles) areas. The convex polygons (farmed: red; ranched: gray) connect the outermost site points of each land‐use type, highlighting the dissimilarity between the avian communities. ANOSIM tests on land‐use type: (a) *R* = .069, *p* = .036; (b) *R* = .056, *p* = .373; (c) *R* = 0.156, *p* = .042; (d) *R* = .223, *p* = .002. Vector lengths are proportional to the correlation between feeding guilds and the two main ordination axes; arrows indicate the direction of change in feeding guild densities

Nonmetric ordination plots show distinct groupings of sites when stratified by land use, suggesting dissimilarities between avian assemblages present on farmed and ranched land (Figure 7). ANOSIM tests confirm that these differences in the bird communities were significant in all habitats combined (top left, *R* = .069, *p* = .036), in miombo woodland (bottom left, *R* = .156, *p* = .042), and open habitat (bottom right, *R* = .223, *p* = .002), but not in acacia woodland (top‐right, *R* = .056, *p* = .373). The feeding guild arrows overlaid onto the ordination plots show predators were more associated with ranched, not farmed, habitats. The arrows also highlight the differential impact of land transformation on avian communities in acacia woodland compared with miombo woodland and open habitats. Whereas the abundance of frugivores, insectivores, nectarivores, and omnivores in miombo and open areas increased in farmed land, the reverse was true of acacia woodland birds. However, *R*‐statistic values (.056–.223) are low, indicating relatively even dissimilarities within and between the land uses (Clarke, 1993).

### 3.1 Functional traits' index responses

Land‐use change from ranched to farmed management appears to have impacted functional traits' indices most strongly in bird communities in acacia woodlands (Table 4). Effect sizes > 1 suggest that ecologically significant changes have occurred in the diversity (decreased), and evenness (increased), of bird traits in farmed acacia woodland that had previously been ranched. There are also weaker indications (ES < 1) of change in the divergence of traits in birds recorded in farmed acacia woodlands (increased) and open habitats (decreased), compared with those ranched habitats.

**Table 4:** Functional traits' indices for bird communities recorded in ranched and farmed sites, with both seasons combined. Effect size (ES) values for each functional index are calculated for the bird communities in the same habitat type, but different land uses

Metric	Acacia woodland		Miombo woodland		Open habitat	
	Ranched	Farmed	Ranched	Farmed	Ranched	Farmed
Functional diversity (FD)	0.739	0.409	0.810	0.755	0.429	0.419
*SD*	0.188	0.152	0.206	0.209	0.114	0.199
Effect size	1.93		0.27		0.06	
Functional divergence (Fdiv)	0.695	0.719	0.689	0.701	0.708	0.660
*SD*	0.033	0.022	0.056	0.032	0.062	0.042
Effect size	0.86		0.26		0.91	
Functional evenness (Feve)	0.470	0.544	0.544	0.539	0.535	0.488
*SD*	0.070	0.036	0.045	0.063	0.061	0.062
Effect size	1.33		0.09		0.76	

Note

Effect sizes that may indicate ecologically significant differences in the distribution of traits within the bird communities are highlighted (blue, ES = 0.8–1.0; red, ES > 1.0).

## 4 DISCUSSION

Land‐use change, sometimes driven by land reform programs, can have substantial impacts on biodiversity (Chappell et al., 2013). We found significant changes (as indicated by effect sizes) in the bird communities in an area where land previously used for cattle and wildlife grazing was converted to arable and mixed livestock farming by smallholders. The effects on species richness, population densities, diversity, and functional trait distribution were complex. Most, but not all, species and functional groups were positively, or neutrally, affected by land‐use change. In farmed open habitats (arable areas and grasslands), and in miombo woodlands, there were increases in species richness, population densities, and biomasses of most feeding guilds. The impact on bird communities in farmed acacia woodland areas that had been ranched was more varied; densities of resident birds increased, but while densities of some feeding guilds increased, others decreased. In both summer and winter, farmed open sites hosted more species than equivalent ranched sites; however, ranched, not farmed, acacia woodlands hosted more species in winter.

The introduction of smallholder mixed farming to previously ranched land may have increased the complexity of the landscape at a spatial scale that benefits most bird species. High landscape complexity in African agroecosystems can maintain species' densities, richness, and abundance in relation to natural habitats (for birds, see Gove, Hylander, Nemomissa, Shimelis, & Enkossa, 2013; for pollinators, see Hagen & Kraemer, 2010; Kasina, 2007; Otieno et al., 2011, but see Gemmill‐Herren & Ochieng, 2008; Sande, Crewe, Raina, Nicolson, & Gordon, 2009). In our study, it thus far appears that these mixed systems, which are representative of a land‐sharing scenario, are allowing bird biodiversity to persist within the agricultural matrix. However, agricultural yields are likely to be low compared to conventional practices, potentially meaning that more land would be required for the same amount of commodity production.

While the effect of land redistribution in this study area was positive based on species richness and diversity, this may be misleading from the perspectives of conservation and functional diversity. Species composition and functional traits of the avian communities differed between sites in different areas of land use. Comparing bird densities by feeding guild in farmed and ranched sites shows that the former supported higher, or similar, densities of most guilds in most habitats. Exceptions were omnivores and predators in acacia woodlands, and nectarivores in miombo woodlands and open habitats. Indeed, guild associations within the NMDS community space showed that there was a different association of guilds following land‐use change in acacia woodland bird communities, compared with other habitats (Figure 6). The differential impact of land‐use change on acacia woodland birds was also highlighted by the functional traits' analyses. Effect sizes indicate that changes in possible ecological significance occurred in the diversity and evenness of their traits, with a smaller effect in traits' divergence. This was likely due to greater number and/or diversity of distinctive large species of omnivores and predators. Indeed, large‐bodied bird species (>150 g) were less abundant in farmed sites, while smaller species (<150 g) were more abundant. Of the larger species, 44% were predators, 33% omnivores, and 11% insectivores. While the majority were Least Concern, White‐backed Vulture (Critically Endangered), and Bateleur *Terathopius ecaudatus* and Kori Bustard (both Near Threatened) are all of conservation concern (IUCN, 2017). Numbers of these three species were low, so evidence for population change was weak. The reduction in large bird species in the farmed sites echoes similar widespread declines across the tropics and in Africa (Newbold et al., 2013). Drivers of these declines include hunting for food (Thiollay, 2006a, 2006b), poisoning (Ogada & Keesing, 2010; Virani, Kendall, Njoroge, & Thomsett, 2011), and habitat loss (Thiollay, 2006a).

A positive association between a species' size and its influence on ecosystem function is usually found, largely because biomass is directly related to the amount of energy and resources assimilated within a species (Grime, 1998; Villéger et al., 2008). Therefore, the impact of the loss of large species may not be captured by abundance‐weighted analyses of community function. For example, one male Kori Bustard weighs approximately as much as 1,500 Yellow‐fronted Canaries, the species that benefited most in terms of increased population density in sites that were transformed to farming. We found that the biomass of different feeding guilds differed in farmed areas and ranched areas, something which might impact ecosystem function (Şekercioğlu, 2006). For example, omnivores were most negatively affected by land‐use change, with reduced or absent populations of four species, namely Swainson's Spurfowl *Pternistis swainsonii*, Helmeted Guineafowl *Numida meleagris*, Shelley's Francolin *Scleroptila shelleyi*, and Kori Bustard. All of these species have varied diets, which include the seeds of indigenous plants, weeds and pioneer grasses, and some crop pests, such as locusts (Urban et al., 1986). These species provide the ecosystem function of seed dispersal as well as being trophic process linkers that impact on populations of invertebrates and small vertebrates (Şekercioğlu, 2006). In addition, the Kori Bustard provides a cultural ecosystem service as it has an iconic status in African culture. White‐backed Vulture and African Fish Eagle were also large species absent from the farmed sites, which had a major impact on estimates of predator biomass. As scavengers, vultures provide a range of ecosystem services, including disease limitation, and maintenance of food‐web energy flow (Şekercioğlu, 2006). While a declining vulture population could have ecosystem consequences, our survey methods were not optimal for large, wide‐ranging species, so drawing firm conclusions is difficult. However, it is unlikely that farming activities were directly attributable to the absence of African Fish Eagle (an obligate piscivore) from farmed sites, but human disturbance may have been a factor.

Despite the loss of large species of potentially high functional and cultural value, the effects of land‐use change from land redistribution on bird communities appear less severe than elsewhere in Africa. For example, agricultural lands bordering the Serengeti reserve had greatly reduced avian species richness, with insectivores and large terrestrial feeders most affected (Sinclair et al., 2002, 2014). Although these Serengeti studies also found a reduction in large‐bodied birds, many of our other findings are in marked contrast to those of Sinclair et al. (2002, 2014). There is a considerable overlap between the avifauna of western Zimbabwe and western Tanzania, and in both study sites, subsistence farming is carried out beside protected areas. In Serengeti however, the protected area is a wildlife conservation reserve, not a ranched area for livestock and game animals. Major differences in methodologies used in the two studies could account for higher abundances of smaller inconspicuous birds in our surveys (entirely walked transects in our study vs. driven transects with stops in Serengeti; no correction for detection probabilities in Serengeti). We also included all birds recorded along farmed transects, not just birds seen within the cultivated or habituated parts. Other important factors are likely to be differences in the natural vegetation, human population density, agricultural intensity, and types of crops grown in the farmed mosaics. The elapsed timescale between the commencement of farming in the area and the study was also at least five times longer in the case of Serengeti (Sinclair et al., 2002). Bird species richness and abundance were lower outside protected areas at three sites across South Africa, and insectivore richness was much higher inside protected areas, with the converse true for granivores (Greve, Chown, Rensburg, Dallimer, & Gaston, 2011). However, the redistributed lands in this Zimbabwean case study are dynamic and young, with relatively low human population density. Conservation of the extensive remaining miombo and acacia woodlands that are now interspersed with arable fields and grasslands in the farmed area will be critical to maintaining biodiversity. Efforts to reduce hunting or persecution of large bird species will also play an important role. Research from other parts of Africa may provide an indication of the future trajectories for Zimbabwe's bird communities; in the absence of conservation interventions, widespread species loss and a decline in abundance across all guilds and body sizes can accompany land‐use changes (Greve et al., 2011; Sinclair et al., 2002, 2014).

## 5 CONCLUSION

Taxonomic measures of bird species richness and diversity were not lower in areas transformed from ranched to farmed land. On the contrary, many measured statistics increased with land transformation, indicating that numerous species and several functional groups have benefited, although a few have not. This may be a temporary effect; only one decade has passed since small‐scale farming commenced on the ranched lands. Changes in the functional traits of birds present in the transformed land suggest that the diversity of traits has reduced, and these are now more evenly distributed across the community. However, our analyses may not have fully captured the paucity of larger species in the farmed sites. Relying on taxonomic measures of diversity and abundance‐weighted functional traits may, therefore, obscure functional changes in bird communities and, by extension, important information required for avian conservation.

It is unknown whether smallholder‐farmed landscapes in the Global South can deliver multiple benefits, for example, in terms of food production and habitat for biodiversity (e.g., Brussaard et al., 2010; Chappell & LaValle, 2011). If we are to facilitate the uptake of biodiversity‐friendly agricultural practices through evidence‐based prioritization, it is essential to quantify the relationships between farming, land management practices, and biodiversity. Similarly, we must recognize the central role that improving income and yields has in a small‐scale farming setting across the developing world. Here, we provide an insight into how avian abundance and diversity differs between newly established small‐scale farms and commercial ranched land. Long‐term monitoring of the avian community is needed to understand the temporal dynamics of change and its driving factors within the context of this land redistribution program.

## CONFLICT OF INTEREST

The authors have no conflicts of interest to declare.

## AUTHOR CONTRIBUTIONS

MD, NC, and PJM conceived and designed the study. MD, NC, and SP collected the data. MD, PRS, and SP analyzed the data. All authors wrote and made substantive revisions to the article and approved the final version for publication.

## Data Availability

Data used in the analyses are accessible from the Research Data Leeds Repository (http://archive.researchdata.leeds.ac.uk/) under citation: Dallimer (2019).
