# Supplementary figures and images for: Rapid redistribution of agricultural land alters avian richness, abundance, and functional diversity
Source: Ecol Evol. 2019 Oct 6;9(21):12259–71. doi: 10.1002/ece3.5713 (PMC6854327; doi:10.1002/ece3.5713)

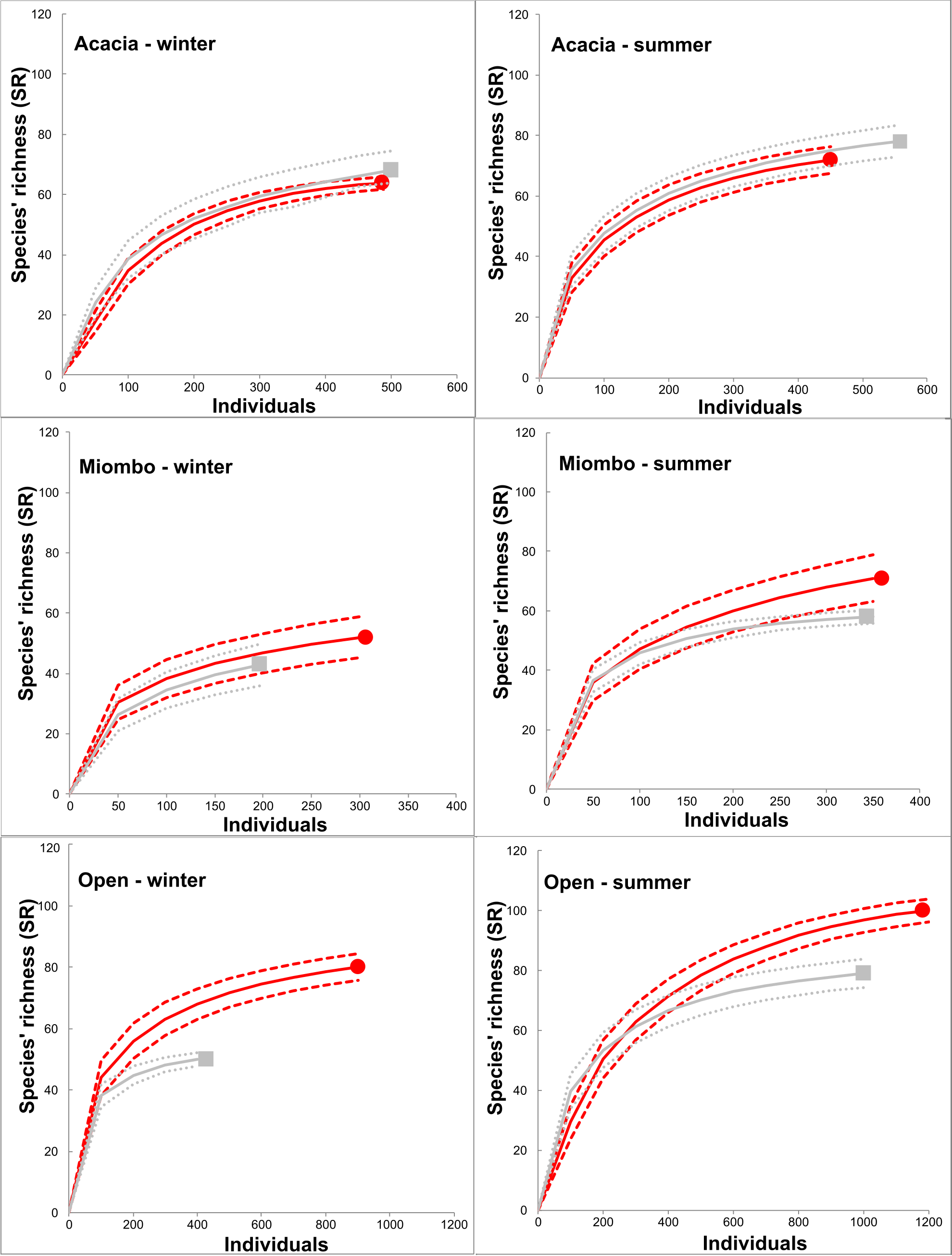

Supplement: Supplementary file 1 [file ECE3-9-12259-s001.png]
