# Supplementary material for: Rapid redistribution of agricultural land alters avian richness, abundance, and functional diversity
Source: Ecol Evol. 2019 Oct 6;9(21):12259–71. doi: 10.1002/ece3.5713 (PMC6854327; doi:10.1002/ece3.5713)
Supplement: Supplementary file 2 [file ECE3-9-12259-s002.docx]

**Appendices**

Table S1. List of bird species recorded across all transects showing primary feeding guilds, migratory behaviour, morphological measurements, and all food types eaten. The naming convention used is the IOC World Bird List v 7.3 (http://dx.doi.org/10.14344/IOC.ML.7.3.)

| **Common species name** | **Scientific species name** | **Feeding**  **Guild** | **Migr**  **Behv** | **Mass**  **(g)** | **Wing**  **(mm)** | **Tail**  **(mm)** | **Bill**  **(mm)** | **Tarsus**  **(mm)** | **Frugivore** | **Granivore** | **Insectivore** | **Nectarivore** | **Predator** |
| --- | --- | --- | --- | --- | --- | --- | --- | --- | --- | --- | --- | --- | --- |
| Acacia Pied Barbet | *Tricholaema leucomelas* | frugivore | Res | 30 | 82 | 49 | 20 | 19 | yes | yes | no | no | no |
| African Cuckoo | *Cuculus gularis* | insectivore | IA | 100 | 211 | 152 | 25 | 20 | no | no | yes | no | no |
| African Fish Eagle | *Haliaeetus vocifer* | predator | Res | 2820 | 559 | 252 | 41 | 85 | no | no | no | no | yes |
| African Goshawk | *Accipiter tachiro* | predator | Res | 356 | 230 | 198 | 17 | 63 | no | no | yes | no | yes |
| African Green Pigeon | *Treron calvus* | frugivore | Res | 231 | 171 | 99 | 13 | 22 | yes | no | no | no | no |
| African Grey Hornbill | *Lophoceros nasutus* | omnivore | Res | 208 | 215 | 192 | 88 | 36 | no | no | no | no | no |
| African Hawk Eagle | *Aquila spilogaster* | predator | Res | 1420 | 440 | 272 | 31 | 95 | no | no | no | no | yes |
| African Hoopoe | *Upupa africana* | insectivore | IA | 53 | 137 | 92 | 49 | 19 | no | no | yes | no | yes |
| African Paradise Flycatcher | *Terpsiphone viridis* | insectivore | Res | 13 | 80 | 165 | 16 | 15 | no | no | yes | no | no |
| African Pipit | *Anthus cinnamomeus* | insectivore | Res | 27 | 87 | 64 | 14 | 26 | no | yes | yes | no | no |
| African Wattled Lapwing | *Vanellus senegallus* | insectivore | Res | 224 | 232 | 99 | 34 | 85 | no | yes | yes | no | no |
| African Yellow White-eye | *Zosterops senegalensis* | insectivore | Res | 11 | 59 | 40 | 10 | 15 | yes | no | yes | no | no |
| Amethyst Sunbird | *Chalcomitra amethystina* | nectarivore | Res | 11 | 64 | 41 | 24 | 16 | yes | no | yes | yes | no |
| Amur Falcon | *Falco amurensis* | predator | Pal | 142 | 233 | 121 | 13 | 30 | no | no | yes | no | no |
| Arrow-marked Babbler | *Turdoides jardineii* | insectivore | Res | 72 | 110 | 108 | 24 | 32 | no | no | yes | no | no |
| Ashy Tit | *Melaniparus cinerascens* | insectivore | Res | 20 | 79 | 59 | 13 | 18 | no | no | yes | no | no |
| Banded Martin | *Riparia cincta* | insectivore | IA | 23 | 128 | 60 | 9 | 12 | no | no | yes | no | no |
| Barn Swallow | *Hirundo rustica* | insectivore | Pal | 20 | 125 | 107 | 8 | 11 | no | no | yes | no | no |
| Barred Wren-Warbler | *Calamonastes fasciolatus* | insectivore | Res | 13 | 59 | 55 | 13 | 21 | no | no | yes | no | no |
| Bar-throated Apalis | *Apalis thoracica* | insectivore | Res | 11 | 52 | 55 | 13 | 20 | no | no | yes | no | no |
| Bateleur | *Terathopius ecaudatus* | predator | Res | 2242 | 527 | 109 | 36 | 73 | no | no | no | no | yes |
| Bearded Woodpecker | *Chloropicus namaquus* | insectivore | Res | 83 | 132 | 67 | 31 | 19 | no | no | yes | no | no |
| Black Cuckooshrike | *Campephaga flava* | insectivore | IA | 34 | 104 | 100 | 15 | 19 | yes | no | yes | no | no |
| Black-backed Puffback | *Dryoscopus cubla* | insectivore | Res | 27 | 80 | 71 | 19 | 22 | no | no | yes | no | no |
| Black-bellied Bustard | *Lissotis melanogaster* | insectivore | Res | 1966 | 353 | 186 | 44 | 131 | no | no | yes | no | no |
| Black-chested Snake Eagle | *Circaetus pectoralis* | predator | Res | 1962 | 510 | 272 | 34 | 87 | no | no | no | no | yes |
| Black-collared Barbet | *Lybius torquatus* | omnivore | Res | 59 | 92 | 57 | 23 | 21 | yes | no | yes | no | no |
| Black-crowned Tchagra | *Tchagra senegalus* | insectivore | Res | 51 | 86 | 101 | 23 | 28 | no | no | yes | no | yes |
| Black-eared Seedeater | *Crithagra mennelli* | granivore | Res | 15 | 81 | 52 | 11 | 13 | no | yes | no | no | no |
| Black-headed Heron | *Ardea melanocephala* | predator | Res | 1078 | 401 | 157 | 100 | 136 | no | no | yes | no | yes |
| Black-headed Oriole | *Oriolus larvatus* | omnivore | Res | 65 | 137 | 97 | 28 | 22 | yes | no | yes | no | no |
| Blacksmith Lapwing | *Vanellus armatus* | insectivore | Res | 156 | 211 | 88 | 28 | 73 | no | no | yes | no | no |
| Black-throated Canary | *Crithagra atrogularis* | omnivore | Res | 11 | 71 | 43 | 9 | 12 | no | yes | yes | no | no |
| Black-winged Kite | *Elanus caeruleus* | predator | Res | 248 | 272 | 122 | 17 | 36 | no | no | yes | no | yes |
| Blue Waxbill | *Uraeginthus angolensis* | granivore | Res | 11 | 52 | 54 | 10 | 14 | no | yes | yes | no | no |
| Bronze Mannikin | *Lonchura cucullata* | granivore | Res | 9 | 49 | 30 | 10 | 14 | no | yes | no | no | no |
| Brown Snake Eagle | *Circaetus cinereus* | predator | Res | 2048 | 514 | 270 | 43 | 100 | no | no | no | no | yes |
| Brown-crowned Tchagra | *Tchagra australis* | insectivore | Res | 33 | 76 | 94 | 18 | 24 | no | no | yes | no | yes |
| Brubru | *Nilaus afer* | insectivore | Res | 24 | 84 | 57 | 16 | 22 | no | no | yes | no | no |
| Bushveld Pipit | *Anthus caffer* | insectivore | Res | 16 | 72 | 53 | 11 | 17 | no | no | yes | no | no |
| Cape Starling | *Lamprotornis nitens* | insectivore | Res | 88 | 132 | 90 | 23 | 34 | yes | no | yes | no | no |
| Cape Wagtail | *Motacilla capensis* | insectivore | IA | 21 | 82 | 84 | 14 | 23 | no | no | yes | no | no |
| Capped Wheatear | *Oenanthe pileata* | insectivore | Res | 33 | 94 | 59 | 15 | 31 | no | no | yes | no | no |
| Cardinal Woodpecker | *Dendropicos fuscescens* | insectivore | Res | 31 | 94 | 47 | 19 | 16 | no | no | yes | no | no |
| Chestnut-backed Sparrow Lark | *Eremopterix leucotis* | granivore | Res | 13 | 83 | 46 | 11 | 16 | no | yes | yes | no | no |
| Chestnut-vented Warbler | *Sylvia subcoerulea* | insectivore | Res | 15 | 66 | 68 | 12 | 21 | yes | no | yes | no | no |
| Chinspot Batis | *Batis molitor* | insectivore | Res | 12 | 60 | 47 | 13 | 18 | no | no | yes | no | no |
| Cinnamon-breasted Bunting | *Emberiza tahapisi* | granivore | Res | 14 | 77 | 60 | 10 | 16 | no | yes | yes | no | no |
| Common Buzzard | *Buteo buteo vulpinus* | predator | Pal | 739 | 369 | 188 | 22 | 75 | no | no | yes | no | yes |
| Common Quail | *Coturnix coturnix* | omnivore | Res | 96 | 105 | 36 | 13 | 24 | no | no | no | no | no |
| Common Scimitarbill | *Rhinopomastus cyanomelas* | insectivore | Res | 37 | 108 | 125 | 42 | 19 | no | no | yes | no | no |
| Common Swift | *Apus apus* | insectivore | Res | 35 | 170 | 72 | 7 | 11 | no | no | yes | no | no |
| Common Waxbill | *Estrilda astrild* | granivore | Pal | 8 | 49 | 56 | 9 | 15 | no | yes | no | no | no |
| Crested Barbet | *Trachyphonus vaillantii* | omnivore | Res | 71 | 102 | 86 | 23 | 26 | no | no | no | no | no |
| Crested Francolin | *Dendroperdix sephaena* | omnivore | Res | 342 | 151 | 95 | 22 | 44 | no | no | no | no | no |
| Crimson-breasted Shrike | *Laniarius atrococcineus* | insectivore | Res | 48 | 99 | 100 | 23 | 32 | no | no | yes | no | no |
| Croaking Cisticola | *Cisticola natalensis* | insectivore | Res | 21 | 66 | 59 | 14 | 28 | no | no | yes | no | no |
| Crowned Lapwing | *Vanellus coronatus* | insectivore | Res | 155 | 202 | 91 | 31 | 68 | no | no | yes | no | no |
| Dark-capped Bulbul | *Pycnonotus tricolor* | frugivore | Res | 39 | 97 | 87 | 17 | 21 | yes | no | no | no | no |
| Diederik Cuckoo | *Chrysococcyx caprius* | insectivore | IA | 32 | 118 | 83 | 17 | 16 | no | no | yes | no | no |
| Emerald-spotted Wood Dove | *Turtur chalcospilos* | granivore | Res | 64 | 111 | 84 | 18 | 18 | yes | yes | no | no | no |
| European Bee-eater | *Merops apiaster* | insectivore | Res | 55 | 148 | 117 | 38 | 11 | no | no | yes | no | no |
| European Roller | *Coracias garrulus* | insectivore | Pal | 140 | 194 | 125 | 36 | 23 | no | no | yes | no | yes |
| Fiery-necked Nightjar | *Caprimulgus pectoralis* | insectivore | Pal | 55 | 161 | 120 | 12 | 16 | no | no | yes | no | no |
| Flappet Lark | *Mirafra rufocinnamomea* | insectivore | IA | 26 | 81 | 55 | 14 | 22 | no | yes | yes | no | no |
| Fork-tailed Drongo | *Dicrurus adsimilis* | insectivore | Res | 51 | 134 | 119 | 21 | 22 | no | no | yes | no | no |
| Freckled Nightjar | *Caprimulgus tristigma* | insectivore | Res | 79 | 190 | 132 | 13 | 19 | no | no | yes | no | no |
| Gabar Goshawk | *Micronisus gabar* | predator | Res | 155 | 195 | 163 | 13 | 45 | no | no | yes | no | yes |
| Golden-breasted Bunting | *Emberiza flaviventris* | granivore | Res | 18 | 82 | 69 | 13 | 17 | no | yes | yes | no | no |
| Golden-tailed Woodpecker | *Campethera abingoni* | insectivore | Res | 68 | 118 | 65 | 27 | 17 | no | no | yes | no | no |
| Great Spotted Cuckoo | *Clamator glandarius* | insectivore | Res | 130 | 193 | 182 | 29 | 29 | no | no | yes | no | no |
| Greater Blue-eared Starling | *Lamprotornis chalybaeus* | frugivore | Res | 86 | 131 | 90 | 19 | 32 | yes | no | no | no | no |
| Greater Honeyguide | *Indicator indicator* | insectivore | Res | 48 | 109 | 70 | 14 | 15 | no | no | yes | no | no |
| Green Wood Hoopoe | *Phoeniculus purpureus* | insectivore | Res | 71 | 154 | 236 | 51 | 22 | no | no | yes | no | no |
| Green-capped Eremomela | *Eremomela scotops* | insectivore | Res | 9 | 57 | 47 | 11 | 18 | no | no | yes | no | no |
| Green-winged Pytilia | *Pytilia melba* | omnivore | Res | 15 | 59 | 49 | 13 | 15 | no | yes | yes | no | no |
| Grey Go-away-bird | *Corythaixoides concolor* | frugivore | Res | 268 | 220 | 245 | 24 | 40 | yes | no | no | no | no |
| Grey Penduline Tit | *Anthoscopus caroli* | insectivore | Res | 6 | 51 | 27 | 8 | 13 | no | no | yes | no | no |
| Grey-backed Camaroptera | *Camaroptera brevicaudata* | insectivore | Res | 11 | 54 | 39 | 12 | 21 | no | no | yes | no | no |
| Grey-headed Bushshrike | *Malaconotus blanchoti* | insectivore | Res | 77 | 114 | 111 | 28 | 32 | no | no | yes | no | yes |
| Grey-headed Kingfisher | *Halcyon leucocephala* | insectivore | Res | 45 | 100 | 57 | 39 | 11 | no | no | yes | no | yes |
| Grey-rumped Swallow | *Pseudhirundo griseopyga* | insectivore | IA | 10 | 97 | 73 | 5 | 11 | no | no | yes | no | no |
| Groundscraper Thrush | *Turdus litsitsirupa* | insectivore | Res | 76 | 128 | 69 | 27 | 33 | no | no | yes | no | no |
| Hamerkop | *Scopus umbretta* | predator | Res | 422 | 305 | 156 | 82 | 70 | no | no | yes | no | yes |
| Helmeted Guineafowl | *Numida meleagris* | omnivore | Res | 1480 | 265 | 171 | 25 | 81 | no | no | no | no | no |
| Jacobin Cuckoo | *Clamator jacobinus* | insectivore | Res | 74 | 157 | 182 | 22 | 25 | no | no | yes | no | no |
| Jameson's Firefinch | *Lagonosticta rhodopareia* | granivore | Res | 9 | 48 | 41 | 10 | 13 | no | yes | no | no | no |
| Klaas's Cuckoo | *Chrysococcyx klaas* | insectivore | IA | 28 | 102 | 74 | 16 | 15 | no | no | yes | no | no |
| Kori Bustard | *Ardeotis kori* | omnivore | Res | 16250 | 678 | 370 | 98 | 206 | no | yes | yes | no | yes |
| Kurrichane Thrush | *Turdus libonyana* | insectivore | Res | 60 | 116 | 97 | 22 | 29 | no | no | yes | no | yes |
| Lanner Falcon | *Falco biarmicus* | predator | Res | 587 | 333 | 183 | 20 | 50 | no | no | yes | no | yes |
| Laughing Dove | *Spilopelia senegalensis* | granivore | Res | 103 | 138 | 110 | 16 | 23 | no | yes | yes | no | no |
| Lesser Grey Shrike | *Lanius minor* | insectivore | Res | 46 | 116 | 89 | 17 | 24 | no | no | yes | no | no |
| Lesser Kestrel | *Falco naumanni* | predator | Res | 159 | 240 | 146 | 13 | 31 | no | no | yes | no | yes |
| Levaillant's Cisticola | *Cisticola tinniens* | insectivore | Pal | 12 | 51 | 55 | 11 | 19 | no | no | yes | no | no |
| Lilac-breasted Roller | *Coracias caudatus* | insectivore | Pal | 106 | 166 | 187 | 33 | 22 | no | no | yes | no | yes |
| Little Bittern | *Ixobrychus minutus* | predator | Res | 148 | 142 | 45 | 48 | 43 | no | no | yes | no | yes |
| Little Sparrowhawk | *Accipiter minullus* | predator | Res | 90 | 150 | 117 | 10 | 42 | no | no | yes | no | yes |
| Long-billed Crombec | *Sylvietta rufescens* | insectivore | Res | 12 | 61 | 28 | 15 | 19 | no | no | yes | no | no |
| Magpie Shrike | *Urolestes melanoleucus* | insectivore | Res | 82 | 134 | 282 | 18 | 33 | no | no | yes | no | yes |
| Malachite Kingfisher | *Corythornis cristatus* | insectivore | Res | 15 | 57 | 27 | 34 | 7 | no | no | yes | no | yes |
| Marico Flycatcher | *Melaenornis mariquensis* | insectivore | Res | 25 | 85 | 76 | 13 | 21 | yes | no | yes | no | no |
| Meyer's Parrot | *Poicephalus meyeri* | frugivore | Res | 117 | 152 | 67 | 20 | 17 | yes | yes | no | no | no |
| Miombo Double-collared Sunbird | *Cinnyris manoensis* | nectarivore | Res | 9 | 63 | 46 | 24 | 17 | yes | no | yes | yes | no |
| Mocking Cliff Chat | *Thamnolaea cinnamomeiventris* | omnivore | Res | 48 | 112 | 95 | 20 | 29 | yes | no | yes | no | no |
| Montagu's Harrier | *Circus pygargus* | predator | Res | 305 | 368 | 221 | 16 | 59 | no | no | yes | no | yes |
| Namaqua Dove | *Oena capensis* | granivore | Res | 40 | 105 | 140 | 14 | 15 | no | yes | no | no | no |
| Natal Spurfowl | *Pternistis natalensis* | omnivore | Pal | 458 | 165 | 96 | 19 | 47 | no | no | no | no | no |
| Neddicky | *Cisticola fulvicapilla* | insectivore | Res | 8 | 48 | 42 | 11 | 17 | no | no | yes | no | no |
| Orange-breasted Bushshrike | *Chlorophoneus sulfureopectus* | insectivore | Res | 27 | 88 | 88 | 16 | 26 | no | no | yes | no | no |
| Pearl-spotted Owlet | *Glaucidium perlatum* | insectivore | Res | 82 | 107 | 76 | 11 | 21 | no | no | yes | no | yes |
| Pied Kingfisher | *Ceryle rudis* | insectivore | Res | 84 | 140 | 74 | 58 | 10 | no | no | yes | no | yes |
| Pin-tailed Whydah | *Vidua macroura* | granivore | Res | 15 | 70 | 130 | 10 | 16 | no | yes | yes | no | no |
| Purple Roller | *Coracias naevius* | insectivore | Res | 168 | 189 | 143 | 41 | 24 | no | no | yes | no | yes |
| Quailfinch | *Ortygospiza atricollis* | omnivore | Res | 11 | 55 | 28 | 9 | 14 | no | yes | yes | no | no |
| Rattling Cisticola | *Cisticola chiniana* | insectivore | Res | 16 | 61 | 60 | 13 | 21 | yes | no | yes | no | no |
| Red-backed Shrike | *Lanius collurio* | insectivore | Pal | 27 | 90 | 76 | 15 | 23 | no | no | yes | no | yes |
| Red-billed Buffalo Weaver | *Bubalornis niger* | insectivore | Res | 81 | 119 | 104 | 23 | 30 | no | no | yes | no | no |
| Red-billed Firefinch | *Lagonosticta senegala* | granivore | Res | 9 | 48 | 36 | 9 | 12 | no | yes | yes | no | no |
| Red-billed Quelea | *Quelea quelea* | granivore | IA | 19 | 66 | 37 | 14 | 18 | no | yes | yes | no | no |
| Red-breasted Swallow | *Cecropis semirufa* | insectivore | IA | 30 | 130 | 118 | 7 | 14 | no | no | yes | no | no |
| Red-capped Lark | *Calandrella cinerea* | insectivore | Res | 24 | 91 | 62 | 13 | 20 | no | yes | yes | no | no |
| Red-chested Cuckoo | *Cuculus solitarius* | insectivore | IA | 73 | 175 | 152 | 22 | 19 | no | yes | yes | no | no |
| Red-collared Widowbird | *Euplectes ardens* | omnivore | Res | 19 | 71 | 130 | 14 | 22 | no | yes | yes | no | no |
| Red-eyed Dove | *Streptopelia semitorquata* | granivore | Res | 235 | 189 | 125 | 22 | 25 | no | yes | yes | no | no |
| Red-faced Mousebird | *Urocolius indicus* | frugivore | Res | 56 | 96 | 210 | 14 | 18 | yes | no | no | no | no |
| Red-headed Weaver | *Anaplectes rubriceps* | insectivore | Res | 22 | 80 | 51 | 17 | 19 | no | no | yes | no | no |
| Red-winged Starling | *Onychognathus morio* | omnivore | Res | 139 | 149 | 126 | 28 | 33 | no | no | no | no | no |
| Retz's Helmetshrike | *Prionops retzii* | insectivore | Res | 48 | 130 | 92 | 24 | 22 | no | no | yes | no | yes |
| Ring-necked Dove | *Streptopelia capicola* | granivore | Res | 153 | 157 | 101 | 13 | 20 | no | yes | yes | no | no |
| Rosy-throated Longclaw | *Macronyx ameliae* | insectivore | Res | 33 | 89 | 79 | 15 | 30 | no | no | yes | no | no |
| Rufous-naped Lark | *Mirafra africana* | insectivore | IA | 42 | 95 | 64 | 20 | 29 | no | yes | yes | no | no |
| Scaly-feathered Weaver | *Sporopipes squamifrons* | granivore | Res | 12 | 57 | 37 | 9 | 15 | no | yes | no | no | no |
| Scarlet-chested Sunbird | *Chalcomitra senegalensis* | nectarivore | Res | 13 | 78 | 43 | 29 | 16 | yes | no | yes | yes | no |
| Secretarybird | *Sagittarius serpentarius* | predator | Res | 4052 | 644 | 700 | 49 | 307 | no | no | yes | no | yes |
| Senegal Coucal | *Centropus senegalensis* | insectivore | Res | 170 | 172 | 205 | 28 | 38 | no | no | yes | no | yes |
| Shelley's Francolin | *Scleroptila shelleyi* | omnivore | Res | 438 | 161 | 79 | 25 | 41 | no | no | no | no | no |
| Shikra | *Accipiter badius* | predator | Res | 123 | 182 | 137 | 11 | 44 | no | no | no | no | yes |
| Southern Black Flycatcher | *Melaenornis pammelaina* | insectivore | Res | 30 | 104 | 93 | 14 | 23 | yes | no | yes | no | no |
| Southern Black Tit | *Melaniparus niger* | insectivore | Res | 22 | 82 | 71 | 11 | 19 | no | no | yes | no | no |
| Southern Fiscal | *Lanius collaris* | insectivore | Res | 39 | 99 | 106 | 20 | 27 | no | no | yes | no | yes |
| Southern Grey-headed Sparrow | *Passer diffusus* | omnivore | Res | 24 | 81 | 61 | 13 | 18 | no | yes | yes | no | no |
| Southern Masked Weaver | *Ploceus velatus* | omnivore | Res | 26 | 76 | 51 | 16 | 21 | no | no | no | no | no |
| Southern Red Bishop | *Euplectes orix* | granivore | Res | 23 | 71 | 40 | 15 | 21 | no | yes | yes | no | no |
| Southern White-crowned Shrike | *Eurocephalus anguitimens* | insectivore | Res | 69 | 136 | 108 | 17 | 24 | yes | no | yes | no | no |
| Southern Yellow-billed Hornbill | *Tockus leucomelas* | omnivore | Res | 190 | 198 | 208 | 64 | 38 | no | no | no | no | no |
| Spotted Flycatcher | *Muscicapa striata* | insectivore | Pal | 15 | 88 | 61 | 13 | 14 | yes | no | yes | no | no |
| Spotted Thick-knee | *Burhinus capensis* | insectivore | Res | 453 | 231 | 123 | 37 | 95 | no | yes | yes | no | no |
| Streaky-headed Seedeater | *Crithagra gularis* | omnivore | Res | 16 | 79 | 59 | 12 | 16 | yes | yes | no | no | no |
| Striped Kingfisher | *Halcyon chelicuti* | insectivore | Res | 38 | 83 | 45 | 32 | 11 | no | no | yes | no | yes |
| Swainson's Spurfowl | *Pternistis swainsonii* | omnivore | Res | 621 | 183 | 84 | 21 | 56 | no | no | no | no | no |
| Tawny-flanked Prinia | *Prinia subflava* | insectivore | Res | 9 | 49 | 61 | 11 | 20 | yes | no | yes | no | no |
| Temminck's Courser | *Cursorius temminckii* | insectivore | IA | 67 | 124 | 46 | 20 | 40 | no | yes | yes | no | no |
| Three-banded Courser | *Rhinoptilus cinctus* | insectivore | Res | 125 | 163 | 83 | 20 | 72 | no | no | yes | no | no |
| Tree Pipit | *Anthus trivialis* | insectivore | Pal | 21 | 86 | 60 | 14 | 21 | no | yes | yes | no | no |
| Tropical Boubou | *Laniarius major* | insectivore | Res | 50 | 95 | 98 | 23 | 34 | no | no | yes | no | yes |
| Village Indigobird | *Vidua chalybeata* | granivore | Res | 12 | 67 | 36 | 8 | 14 | no | yes | no | no | no |
| Village Weaver | *Ploceus cucullatus* | insectivore | Res | 37 | 85 | 54 | 20 | 21 | no | no | yes | no | no |
| Violet-backed Starling | *Cinnyricinclus leucogaster* | frugivore | IA | 45 | 107 | 60 | 15 | 20 | yes | no | yes | no | no |
| Violet-eared Waxbill | *Uraeginthus granatinus* | granivore | Res | 12 | 57 | 66 | 11 | 16 | no | yes | yes | no | no |
| Wahlberg's Eagle | *Hieraaetus wahlbergi* | predator | IA | 1147 | 421 | 219 | 26 | 76 | no | no | no | no | yes |
| White-backed Vulture | *Gyps africanus* | predator | Res | 5380 | 610 | 258 | 48 | 104 | no | no | no | no | yes |
| White-bellied Sunbird | *Cinnyris talatala* | nectarivore | Res | 7 | 52 | 33 | 20 | 16 | yes | no | yes | yes | no |
| White-breasted Cuckooshrike | *Coracina pectoralis* | insectivore | Res | 58 | 141 | 112 | 19 | 23 | no | no | yes | no | no |
| White-browed Scrub Robin | *Cercotrichas leucophrys* | insectivore | Res | 17 | 68 | 65 | 15 | 24 | yes | no | yes | no | no |
| White-browed Sparrow-Weaver | *Plocepasser mahali* | omnivore | Res | 41 | 103 | 63 | 17 | 26 | no | yes | yes | no | no |
| White-crested Helmetshrike | *Prionops plumatus* | insectivore | Res | 33 | 107 | 85 | 20 | 21 | no | no | yes | no | no |
| White-necked Raven | *Corvus albicollis* | omnivore | Res | 911 | 403 | 182 | 63 | 75 | no | no | no | no | yes |
| White-throated Robin-Chat | *Cossypha humeralis* | insectivore | Res | 21 | 78 | 70 | 16 | 27 | yes | no | yes | no | no |
| White-winged Widowbird | *Euplectes albonotatus* | granivore | Res | 21 | 71 | 61 | 14 | 19 | no | yes | yes | no | no |
| Willow Warbler | *Phylloscopus trochilus* | insectivore | Pal | 9 | 66 | 49 | 10 | 18 | no | no | yes | no | no |
| Wire-tailed Swallow | *Hirundo smithii* | insectivore | Res | 12 | 107 | 67 | 8 | 7 | no | no | yes | no | no |
| Yellow-bellied Greenbul | *Chlorocichla flaviventris* | omnivore | Res | 39 | 101 | 96 | 19 | 23 | no | no | no | no | no |
| Yellow Bishop | *Euplectes capensis* | granivore | Res | 19 | 73 | 55 | 19 | 24 | no | yes | yes | no | no |
| Yellow-fronted Canary | *Crithagra mozambica* | omnivore | Res | 11 | 69 | 41 | 9 | 13 | no | yes | yes | no | no |
| Yellow-fronted Tinkerbird | *Pogoniulus chrysoconus* | omnivore | Res | 13 | 62 | 34 | 13 | 13 | yes | no | yes | no | no |
| Yellow-throated Longclaw | *Macronyx croceus* | insectivore | Res | 48 | 101 | 76 | 18 | 35 | no | no | yes | no | no |
| Yellow-throated Petronia | *Gymnoris superciliaris* | omnivore | Res | 25 | 91 | 57 | 14 | 19 | no | yes | yes | no | no |
| Zitting Cisticola | *Cisticola juncidis* | insectivore | Res | 9 | 51 | 38 | 10 | 18 | no | no | yes | no | no |

#
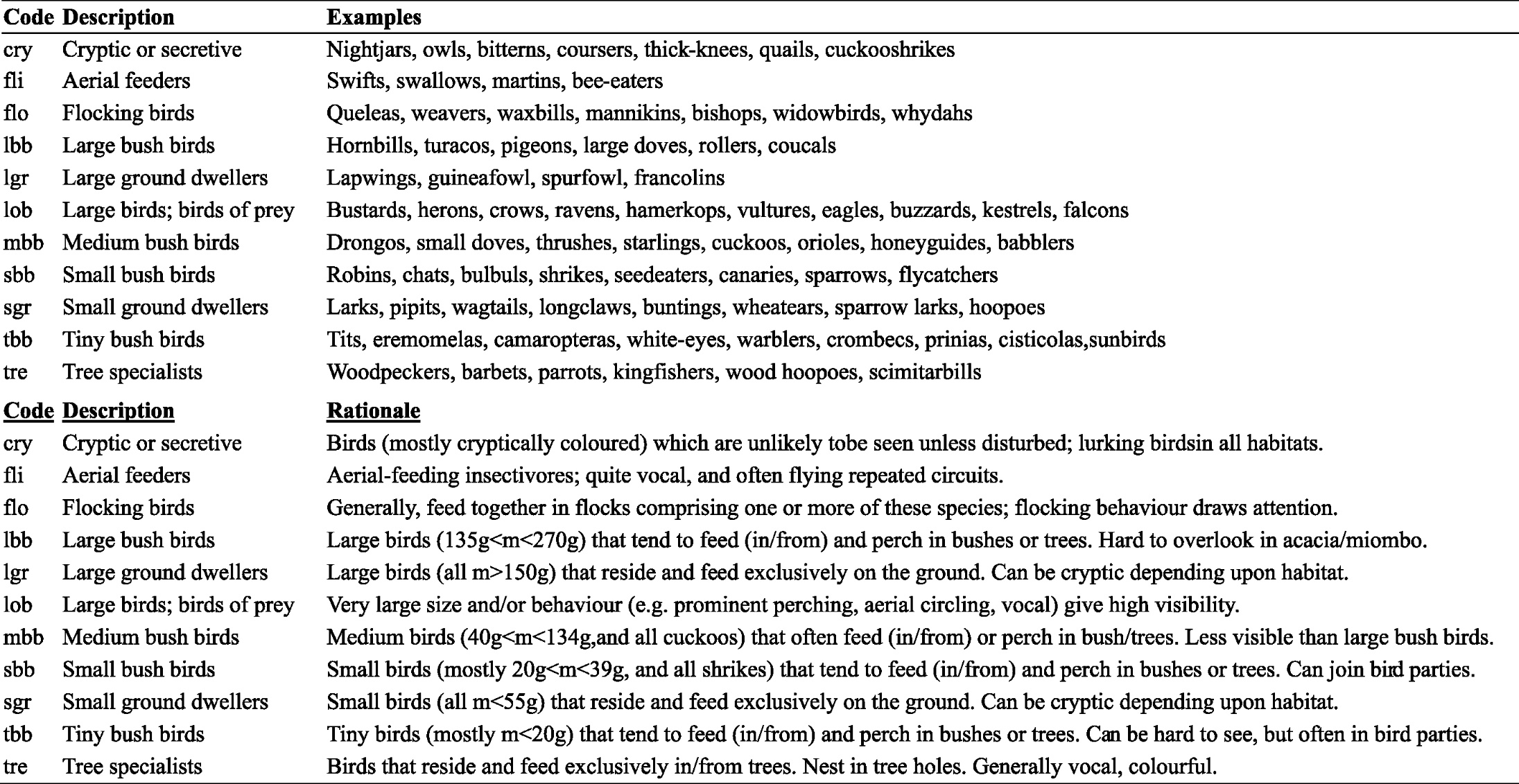
Table S2. Categories and definitions of prominence codes assigned to bird species recorded across all habitats. Each species was classified into a category of prominence, based on our extensive field experience of observing birds in the African savannah.


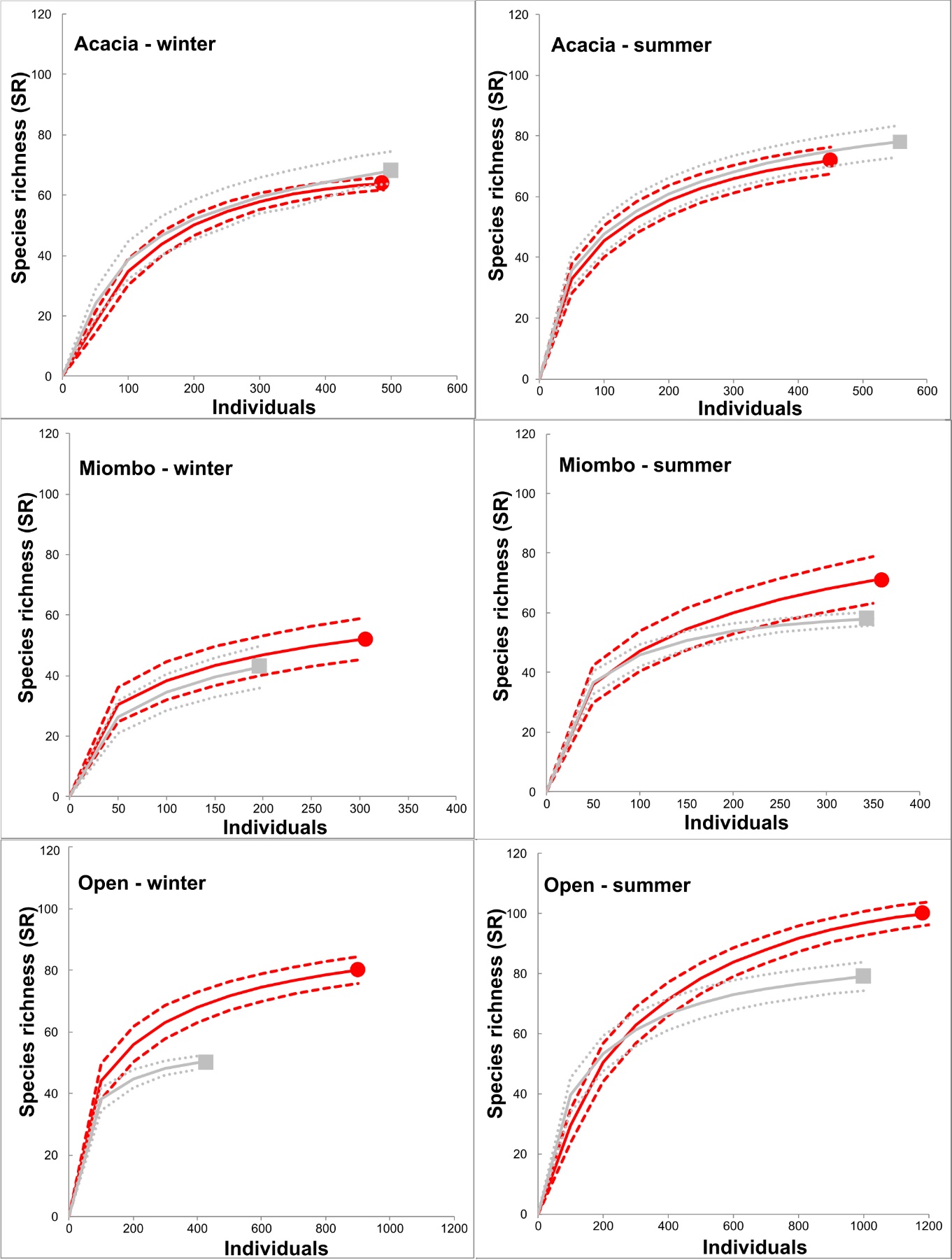


Figure S1. Numbers of birds and species richness (SR) recorded in ranched (grey squares) and farmed sites (red circles) for each habitat and season. The curves are individual-based interpolation (rarefaction) estimates of SR calculated from count data using EstimateS V9.1.0 software (Colwell 2013). Dotted grey (ranched) and red dashed (farmed) lines represent 95% confidence intervals for SR based on unconditional estimates of variances.


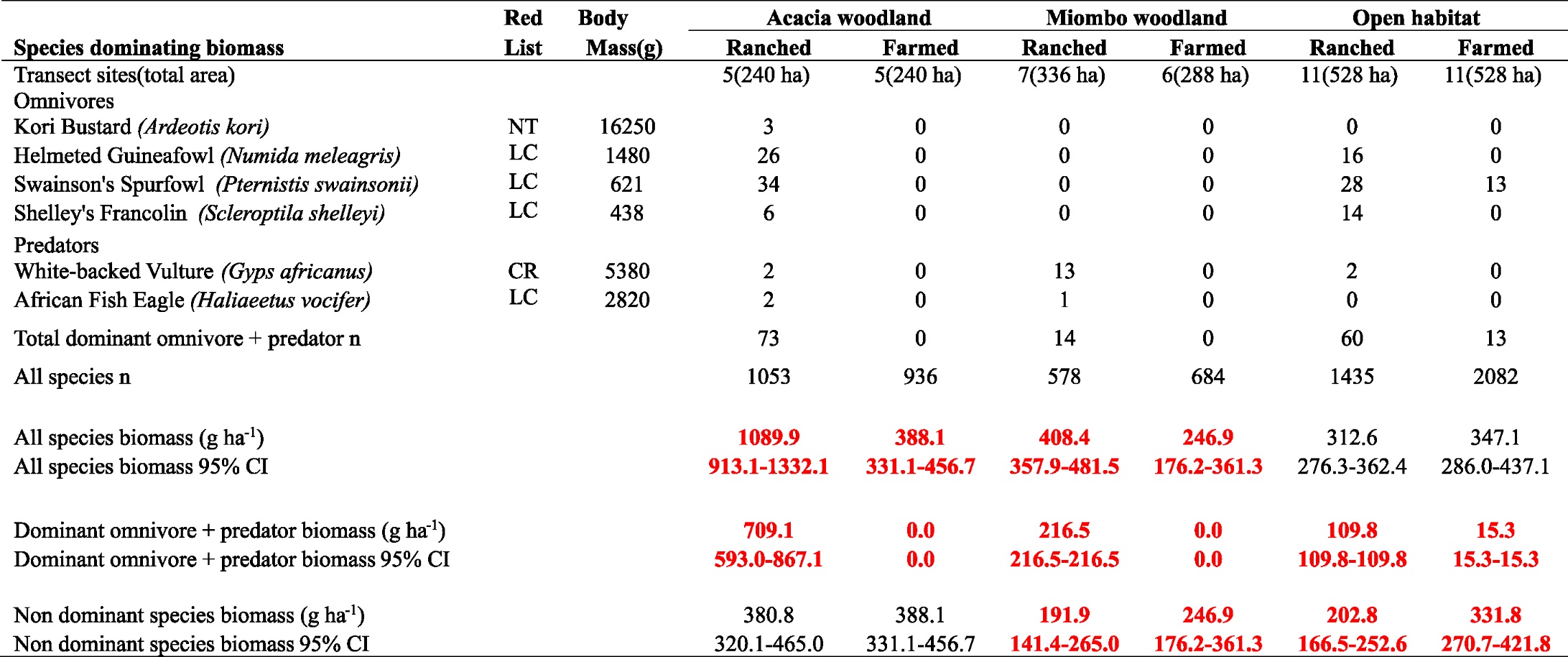
Table S3. Avian biomass in all habitats, for winter and summer combined, showing the effect of small numbers of large species on total biomass. No large species were present among the frugivores, granivores, insectivores and nectarivores. Red indicates an effect size > 1 in the difference between estimated avian biomasses in each biomass category for ranched versus farmed sites of the same habitat type.
